# Differential Transcription of Bacteriophage φX174 Genes at 37°C and 42°C

**DOI:** 10.1371/journal.pone.0035909

**Published:** 2012-04-23

**Authors:** Luyi Zhao, Amber D. Stancik, Celeste J. Brown

**Affiliations:** Department of Biological Sciences, University of Idaho, Moscow, Idaho, United States of America; Naval Research Laboratory, United States of America

## Abstract

To investigate how high temperature affects viral transcription, the absolute amounts of mRNA for six bacteriophage φX174 genes were compared at 37°C and 42°C using Q-PCR. At 37°C, mRNA levels for all genes were consistent with previous studies, but at 42°C mRNA levels for four genes were significantly different from levels at 37°C. Transcript levels were higher for genes *B* and *D*; the promoter before gene *B* appears to be up-regulated at high temperature. Levels for genes *F* and *G* were reduced at high temperature, possibly due to increased efficiency of the transcription termination signal immediately upstream of gene *F*. These functional changes in φX174 gene regulation at high temperature have not been described previously. Studies of phage evolution at high temperatures indicate that this difference in transcript levels is subject to adaptation.

## Introduction

**Figure 1:**
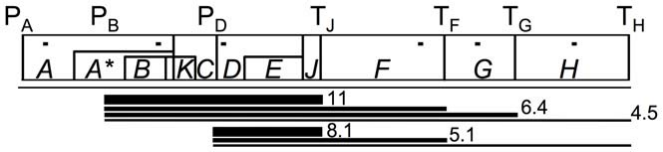
Genetic map of φX174 based on refs. 1 and 2. Note that the map is linearized for clarity; φX174 has a 5,386 nt circular genome. **P** indicates promoter; **T** indicates terminator. **A** to **K** indicate genes. Short bars on the genomic map indicate approximate location of Q-PCR primers for each of six genes. Below the map, bars indicate the transcripts found at 37°C, and numbers indicate transcript half-life in minutes [9].

During the latter half of the twentieth century, the bacteriophage φX174 was used as a model organism for the study of many molecular processes, including DNA replication, viral infection, capsid assembly and genome organization [1], [2]. In addition, φX174 gene expression was well-studied in its host *Escherichia coli* at 37°C. The results of these studies indicate that φX174 recruits host-encoded *trans*-acting proteins and encodes a *cis*-regulatory network composed of three promoters and four terminators (Figure 1). The three promoters, P_A_, P_B_, and P_D_, are found upstream of genes *A*, *B* and *D*, respectively, and are bound by host sigma factors and RNA polymerase to initiate transcription [3], [4], [5]. All genes transcribed from P_D_ are also found on transcripts starting from P_B_. Four terminators, T_J_, T_F_, T_G_ and T_H_, are located downstream of genes *J*, *F*, *G* and *H*, respectively, and are involved in *rho*-independent termination of transcription [6], [7], [8]. The terminators are not stringent, so a fraction of RNA polymerase complexes continue through these termination sites to make longer transcripts. Hayashi and colleagues found that transcript decay rates vary [9]; transcripts that begin at P_B_ or P_D_ and end at T_J_ are more stable than those ending after genes *F*, *G* and *H* (Figure 1). Disruption of the terminator structure of T_J_ accelerates the rate of mRNA degradation of the shorter transcripts [7].

Gene transcription in *E. coli* is affected by temperature due to both enzyme kinetics and the heat shock response [10], [11], [12]. Because φX174 transcription relies heavily on the host, changes that affect host transcription will also affect the phage. However, the effects of temperature on differential φX174 gene expression have not been rigorously determined. Here we demonstrate that the pattern of φX174 gene transcription is different at 42°C, providing new information about *cis*-regulatory regions of this well-studied phage.

## Results and Discussion

Using Q-PCR, the absolute mRNA levels of six genes were compared between 37°C or 42°C infections. There was considerable variation in the total amount of phage mRNA among replicates due to differences in the number of infected cells (total phage mRNA ranged from 0.03 to 1 pg/µl; between these extremes, the average was ∼0.5 pg/µl). Each replicate experiment was done at both temperatures, and the variability between temperatures within a replicate was quite low. To account for between-replicate variation in infection rate, statistical tests were conducted on the *proportion* that each gene contributed to the total phage mRNA within a sample. Note from Figure 1 that each primer amplifies cDNA from multiple transcripts of different lengths, and Q-PCR does not distinguish among these transcripts. Thus the primers for gene *D* amplify all transcripts and the *F* primer set amplifies only those transcripts that read through T_J_.

**Figure 2:**
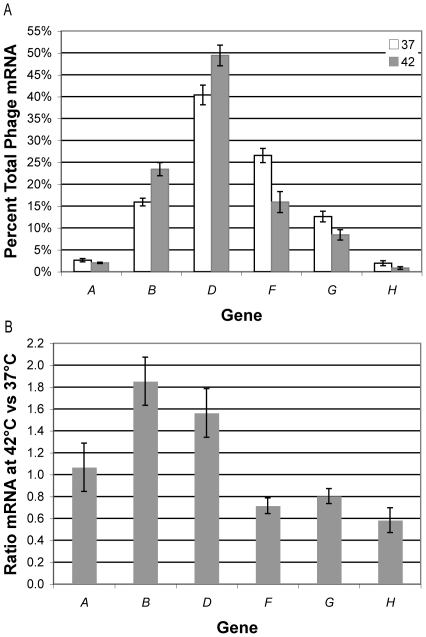
Transcript levels of six φX174 genes measured 4 min post ejection. A. Proportion (%) each gene contributed to the total phage mRNA in a sample averaged over six samples for growth at 37°C and 42°C. Error bars are ±1 standard error. B. Ratio of the absolute quantity of each gene at 42°C to 37°C averaged over six samples. Error bars are ±1 standard error.

Qualitatively, our results at 37°C (Figure 2A) were consistent with the previously published results shown in Figure 1 [1], [2]. Transcript from P_A_ is low due in large part to mRNA instability [9]. There is more mRNA for gene *D* than gene *B* due to P_D_, and there is successively less mRNA for the genes downstream of gene *J* due to the four terminators. There were important differences between the results at 37°C and those at 42°C (Figure 2A). The mRNA levels were greater for genes *B* and *D* at 42°C than at 37°C (df = 1, p-value<0.005), but were less for genes *F* and *G* at 42°C than at 37°C (df = 1, p-value<0.05). Differences in mRNA levels for genes *A* and *H* were not significantly different between temperatures, although there appears to be a two fold reduction in transcript for gene H at the higher temperature. The transcript levels for genes A and H were at the lower limit of detection for our Q-PCR assay, leading to greater variability in their estimates and lower statistical power.

**Figure 3:**
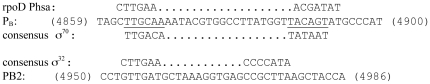
Sequence alignment of sigma factor binding sites. Top: Alignment of the experimentally-determined binding sites for P_B_ [3] (underlined) with the σ^32^ binding sites from the *E. coli rpoD* promoter and with the canonical σ^70^ binding sites [13], [30]. Bottom: Potential heat shock promoter (PB2) downstream of P_B_ aligned with the consensus σ^32^ binding sites [14]. Numbers indicate start and end position of the φX174 sequence, and a dot indicates spacing to maintain alignment.

The amount of mRNA for genes *B* and *D* were greater at 42°C than at 37°C, suggesting that at least one promoter behaves in a manner similar to a heat shock promoter (Figure 2B) [10]. The absolute amount of mRNA for gene *B* is on average 85% greater at 42°C than at 37°C. The increase in the absolute amount of gene *D* mRNA (56%) at 42°C can be accounted for by the increase in transcription from P_B_. Our data reveal that P_B_ responds to high temperature by up-regulating gene expression. This response is like that of a heat shock promoter, which is regulated by the heat shock sigma factor, σ^32^. A sequence with similarity to the −10 and −35 σ^32^ binding sites of the heat shock promoter of the *rpoD* gene overlaps the σ^70^ binding site identified *in vitro* for P_B_ (Figure 3) [3], [13], [14]. These sequence motifs are completely conserved among 16 phages of the phiX clade sampled from nature [15]. There is also another sequence downstream of P_B_ that is similar to the consensus σ^32^ binding site and is also conserved within the phiX clade (Figure 3). These two potential σ^32^ binding sites have low similarity to the consensus σ^32^ site, coinciding with the weak increase in transcription at higher temperature. The apparent importance of P_B_ at high temperature has not been reported previously.

The increase in transcript levels for genes *B* and *D* appear to be biologically as well as statistically significant. Previous studies in which φX174 was evolved in chemostats to investigate adaptation of the phage to high temperature resulted in adaptive substitutions in P_D_ [16], [17], [18]. Our previous work on these substitutions indicate that they down-regulate gene expression at both 37°C and 42°C, virtually eliminating the bottom set of transcripts in Figure 1 [19]. Phage differing from the wild type at any one of these adaptive substitutions have greater doublings per hour than wild type in batch cultures growing at 42°C and can outcompete the wild type phage in a chemostat at 42°C but not at 37°C. Thus there is strong selection to decrease the levels of transcripts for genes after P_D_, indicating that the increase in transcription at 42°C impacts the biology of the phage.

The absolute amounts of mRNA for genes *F* and *G* were on average 28% and 20% less, respectively, at 42°C than at 37°C (Figure 2B). Although this is a relatively small decrease between the two temperatures, it represents a large decrease in the total amount of mRNA that is transcribed from P_B_ and P_D_ at 42°C. Whereas 68% of transcripts read through T_J_ at 37°C, only 34% of transcripts read through at 42°C (Figure 2A). This substantial drop in the absolute amount of gene *F* transcript relative to gene *D* may be explained by the increased termination efficiency of T_J_ at high temperature. The termination efficiency of *rho*-independent transcription terminators is determined by competition between the rates of elongation and termination [12]. At increasing temperature, mRNA synthesis increases linearly due to enzyme kinetics [11]. Counteracting the increased elongation rate, the ternary complex including DNA, mRNA and RNA polymerase may dissociate faster at higher temperature when disrupted by a strong terminator. This effect on translation efficiency is not seen for T_F_ and T_G_ possibly due to the weak structural stability of their hairpin structures. Computationally determined hairpin scores for each terminator, T_J_, T_F_, T_G_ and T_H_, are −9.5, −2.6, −2.1 and −13.2, respectively, where smaller values indicate more stable hairpin structures [20]. Transcription terminators both terminate transcription and stabilize the upstream transcript [21]. The lower structural stability of T_F_ and T_G_ is also suggested by the shorter half-lives of transcripts that end at T_F_ and T_G_ than those that end at T_J_ (Figure 1). Our results illustrate the increased efficiency of the *rho*-independent transcription terminator T_J_ at high temperature.

Visualization of the B, D, F, G and H proteins on SDS gels show that these differences in mRNA levels are not reflected by differences in protein levels (data not shown). mRNA and protein levels are not always correlated in bacteria, and this is particularly true at increased temperatures where mRNA secondary structure and subsequent accessibility of ribosome binding sites may change [22], [23], [24]. We speculate that the transcript level differences at high temperature may be necessary to maintain the ratio of proteins involved in capsid assembly as has been found in phages T4 and lambda [25], [26]. In particular, protein B is stabilized when bound to the coat protein, otherwise, it degrades rapidly [27]. The amount of protein B seen in the gels therefore may be determined by the amount of F protein. Similarity in the ratio of proteins at the two temperatures suggests that adaptive changes that down-regulate transcription may affect some other aspect of phage biology, possibly genome replication rate. Understanding these two possibilities is the direction of our current research program.

Although the biology of φX174 is well-characterized, the functions of its regulatory regions at high temperature have not been fully described. Comparing gene expression at 37°C and 42°C shows that growth at high temperature alters transcription in two ways. Transcript levels were up-regulated from P_B_ as if it were a heat shock promoter and down-regulated for several genes possibly due to increased efficiency of T_J_. These results indicate that there is still more to learn about the regulation of gene expression of φX174 and its related microvirid phage.

## Materials and Methods

To obtain RNA, replicate cultures were grown at either 37°C or 42°C using our previously developed protocol [19]. Briefly, wild-type φX174 (GenBank no. AF176034) infections were synchronized by incubating 1.5×10^8^ *E. coli* C cells with 1.5×10^7^ phages in 1.0 mL of phage Luria-Bertani medium [18] containing 2.0 mM CaCl_2_ at 15°C for 1 hr. Unattached phage were removed by centrifuging at 13000 rpm for 5 minutes and discarding the supernatant. The pellet was resuspended in 1 mL cold medium and added to 9 mL medium pre-warmed to 37°C or 42°C. Samples were taken at 4 min, which is less than the half-life of most transcripts [9]; and added to RNAprotect™ Bacteria Reagent (Qiagen Cat. No. 76506). Total RNA was purified following the RNeasy Mini Kit (Qiagen Cat. No. 74104). RNase-Free DNase Set (Qiagen Cat. No. 79254) was used for on-column DNA digestion. The *E. coli* C strain and the wild type φX174 phage were kindly provided by HA Wichman, University of Idaho [17].

Q-PCR was used to determine the absolute amount of mRNA for each of the six genes. Compared with the method used by Hayashi [28], Q-PCR is more sensitive and accurate, although it does not provide information about individual transcripts. cDNA was synthesized from each mRNA sample, and mRNA levels were detected for genes *A*, *B*, *D*, *F*, *G* and *H* using the protocol described previously and the primer sets listed in Table 1 [19]. Additionally, solutions of purified phage DNA at known concentrations (0.002, 0.0002, 2E-05, 2E-06 ng/µL) were used to determine a standard curve relating absolute mRNA quantity to the cycle threshold (the number of cycles required for the fluorescent signal to cross a threshold value). This standard curve was used to estimate the total amount of mRNA in each sample for each gene.

**Table 1:** Oligonucleotide primers used in Q-PCR of φX174 genes.

Primer Name*	Location**	Sequence (5′ to 3′)
pA_F	4188–4212	GGTGATATGTATGTTGACGGCCATA
pA_R	4309–4333	GGGCGGTGGTCTATAGTGTTATTAA
pA_M	4216–4232	ACGAACGTCAGAAGCAG
pB_F	5337–5360	CTCAAATTTATGCGCGCTTCGATA
pB_R	5377–5397	TTCTGCGTCATGGAAGCGATA
pB_M	27–42	CCAACCTGCAGAGTTT
pD_F	400–424	TTACTGAACAATCCGTACGTTTCCA
pD_R	457–466	ACGGCAGAAGCCTGAATGAG
pD_M	427–443	CCGCTTTGGCCTCTATT
pF_F	1778–1801	CAGTTTTCTGGTCGTGTTCAACAG
pF_R	1839–1863	GCAAGAGTAAACATAGTGCCATGCT
pF_M	1820–1836	CCGCGTTTCTTTGTTCC
pG_F	2611–2635	GTTTCTGTTGGTGCTGATATTGCTT
pG_R	2662–2685	AGAAGACTCAAAGCGAACCAAACA
pG_M	2642–2656	CCGACCCTAAATTTT
pH_F	3318–3333	GCTTGGGAGCGTGCTG
pH_R	3467–3484	CGTGAAGTCGCCGACTGA
pH_M	3451–3466	ATGCCAGCAATCTCTT

* Amplified gene designated by letter before underscore; F and R after underscore indicate forward and reverse amplification primers; M designates labeled probe.

** Numbering based upon GenBank accession AF176034.

To account for between-replicate variation in infection rate, statistical tests were conducted on the proportion that each gene contributed to the total phage mRNA within a sample. A standard variance-stabilizing transformation (arcsin(√p)) was used on the proportions prior to performing the statistical tests [29]. A linear model testing the effect of gene, temperature and the gene-by-temperature interaction on the transformed proportions indicated that gene (F = 268.25, df = 5, p-value<0.0001) and gene-by-temperature interaction (F = 10.37, df = 5, p-value<0.0001) were significant. Thus, each gene was tested separately for the difference in expression between the two temperatures, and p-values were adjusted for multiple tests on the same data using a Bonferroni correction.
